# Unravelling the Polytoxicology of Chlorfenapyr on Non-Target HepG2 Cells: The Involvement of Mitochondria-Mediated Programmed Cell Death and DNA Damage

**DOI:** 10.3390/molecules27175722

**Published:** 2022-09-05

**Authors:** Yuanhang Ren, Xuan He, Xiyue Yan, Yanting Yang, Qiang Li, Tian Yao, Lidan Lu, Lianxin Peng, Liang Zou

**Affiliations:** 1 Key Laboratory of Coarse Cereal Processing of Ministry of Agriculture and Rural Affairs, Chengdu 610106, China; 2 Sichuan Engineering and Technology Research Center of Coarse Cereal Industralization, Chengdu 610106, China; 3 College of Food and Biological Engineering, Chengdu University, Chengdu 610106, China

**Keywords:** Chlorfenapyr, HepG2 cells, apoptosis, autophagy, DNA damage, cell cycle

## Abstract

Chlorfenapyr (CHL) is a type of insecticide with a wide range of insecticidal activities and unique targets. The extensive use of pesticides has caused an increase in potential risks to the environment and human health. However, the potential toxicity of CHL and its mechanisms of action on humans remain unclear. Therefore, human liver cells (HepG2) were used to investigate the cytotoxic effect and mechanism of toxicity of CHL at the cellular level. The results showed that CHL induced cellular toxicity in HepG2 cells and induced mitochondrial damage associated with reactive oxygen species (ROS) accumulation and mitochondrial calcium overload, ultimately leading to apoptosis and autophagy in HepG2 cells. Typical apoptotic changes occurred, including a decline in the mitochondrial membrane potential, the promotion of Bax/Bcl-2 expression causing the release of cyt-c into the cytosol, the activation of cas-9/-3, and the cleavage of PARP. The autophagic effects included the formation of autophagic vacuoles, accumulation of Beclin-1, transformation of LC3-II, and downregulation of p62. Additionally, DNA damage and cell cycle arrest were detected in CHL-treated cells. These results show that CHL induced cytotoxicity associated with mitochondria-mediated programmed cell death (PCD) and DNA damage in HepG2 cells.

## 1. Introduction

Chlorfenapyr (CHL) is a commonly used insecticide worldwide. It is widely used to control Lepidoptera, Diptera, Coleoptera, Hemiptera, and mites on various crops and trees, with good effects [1]. CHL is a type of novel N-substituted halogenated pyrrole compound. It can interrupt the conversion of adenosine diphosphate to adenosine triphosphate in the mitochondria and cause energy loss [2], which eventually leads to cell dysfunction and death.

In light of the popularity of CHL, the potential risks that it poses to the environment and non-target organisms have attracted researchers’ attention in recent years. Studies have shown that CHL binds strongly to soil particles with low water solubility and volatility. The degradation of CHL in soil, sediment, and water is slow, with an average half-life of 1.0 year, 1.1 years, and 0.8 years, respectively. Moreover, the toxic effects of CHL have been reported in ducks, fish, silkworm, and mice [3,4,5,6]. Due to its high environmental persistence and potential toxic effects on non-target organisms, CHL has been banned or limited to use in the Europe and United States [4]. As for humans, a few cases of fatal toxicity of CHL have also been reported. The characteristic features of CHL intoxication are high fever and rhabdomyolysis, which gradually worsen until death [7,8]. Previous studies showed that CHL ingestion causes damage to high-energy organs, such as the brain, kidneys, muscles, and heart. CHL also induces delayed injury, such as Leigh’s disease or mitochondrial neurogastrointestinal encephalopathy, ultimately resulting in death [9].

Considering the toxicity and the widespread use of CHL, non-target organisms, particularly human health, may face potential threats. Therefore, it is vital to re-evaluate the toxic effects and explore the mechanism of action of CHL in humans. In this study, a frequently used cellular model, human liver cells (HepG2), was employed to evaluate the toxic effects of CHL in vitro. Furthermore, multiple tests were performed to explore the mechanism of CHL toxicity in HepG2 cells. We found that CHL inhibited cell proliferation associated with mitochondria-mediated PCD and oxidative DNA damage in HepG2 cells. These findings provide a theoretical basis for the toxic effects of CHL and place a focus on the latent human health and environmental security threats posed by N-substituted halogenated pyrrole insecticides.

## 2. Results

### 2.1. Cytoactivity of HepG2 Cells

**Figure 1:**
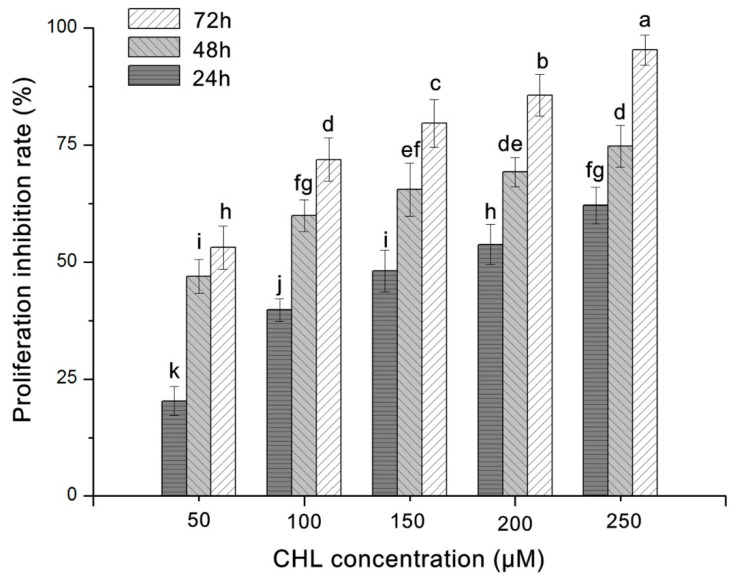
Cytoactivity of chlorfenapyr (CHL) against HepG2 cells under various concentrations at various lengths of time. Distinct letters above the columns indicate significant differences at *p* ≤ 0.05.

The cytoactivity of HepG2 cells treated with increasing concentrations of CHL for 24, 48 and 72 h was assessed by MTT assay. The results showed that CHL inhibited the proliferation of HepG2 cells in a time- and dose-dependent manner (Figure 1). The IC_50_ values of CHL treatment at 24, 48 and 72 h were 161.74, 59.16 and 32.49 μM, respectively.

### 2.2. Production of Reactive Oxygen Species (ROS) and Damage of Mitochondria

**Figure 2:**
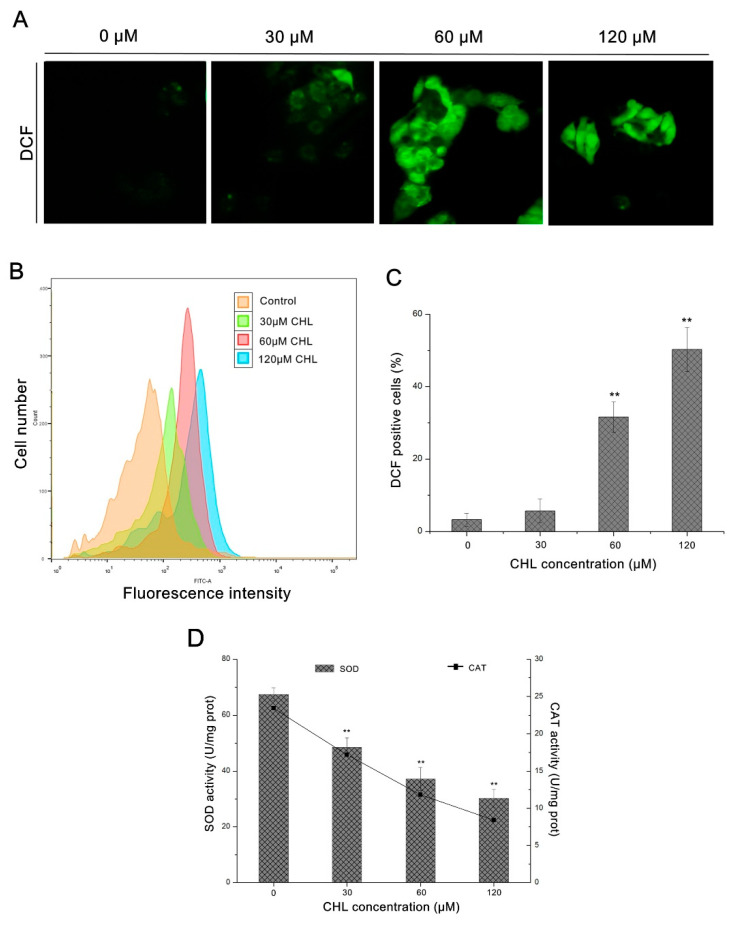
Analysis of ROS production and antioxidant enzyme activity in HepG2 cells after treatment with CHL. (**A**): Analysis of DCF staining by epifluorescence (200×). (**B**): Analysis of DCF staining by flow cytometry. (**C**): Quantification of ROS levels. (**D**): Activity of SOD and CAT enzymes. ** *p* ≤ 0.01 vs. the negative control.

As shown in Figure 2A,B, a gradual increase in dichlorofluorescein (DCF) fluorescence was detected in cells with increasing concentrations of CHL. Compared to the control, the number of DCF-positive cells rose to 51.8 ± 6.1% at 120 μM CHL treatment (Figure 2C). These data suggest that CHL induces ROS generation in HepG2 cells. Additionally, as indicated in Figure 2D, the activity of intracellular antioxidant enzymes SOD and CAT decreased in a dose-dependent manner in cells after treatment with CHL.

**Figure 3:**
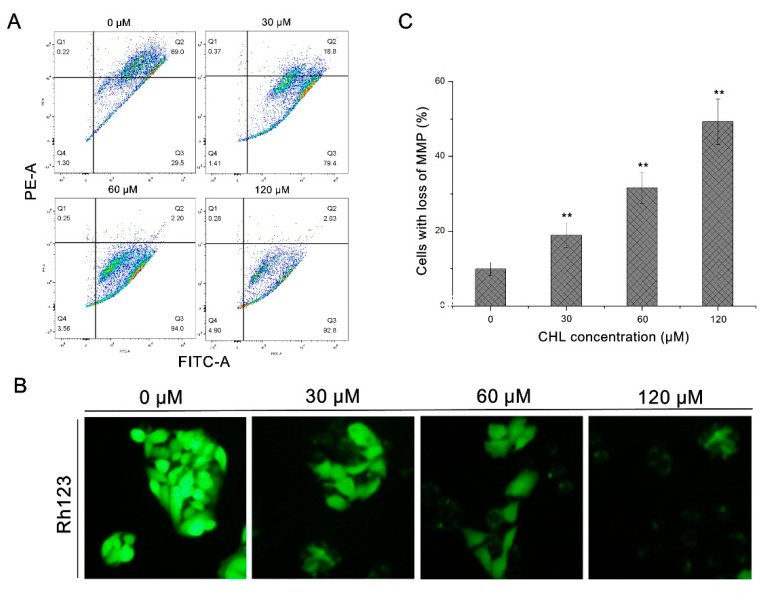
Analysis of MMP loss in HepG2 cells after treatment with CHL. (**A**): Analysis of JC-1 staining by flow cytometry. (**B**): Analysis of Rh123 staining by epifluorescence (200×). (**C**): Quantification levels of MMP loss. ** *p* ≤ 0.01 vs. the negative control.

As demonstrated in Figure 3B, the dose-related decrease in Rh123 fluorescence intensity demonstrated the depolarization of the mitochondrial membrane potential (MMP). Furthermore, after JC-1 staining, we detected that exposure to 120 μM CHL resulted in a 49.3 ± 2.5% decrease in MMP compared to the control (Figure 3A,C). These data suggest that CHL causes MMP collapse in HepG2 cells.

**Figure 4:**
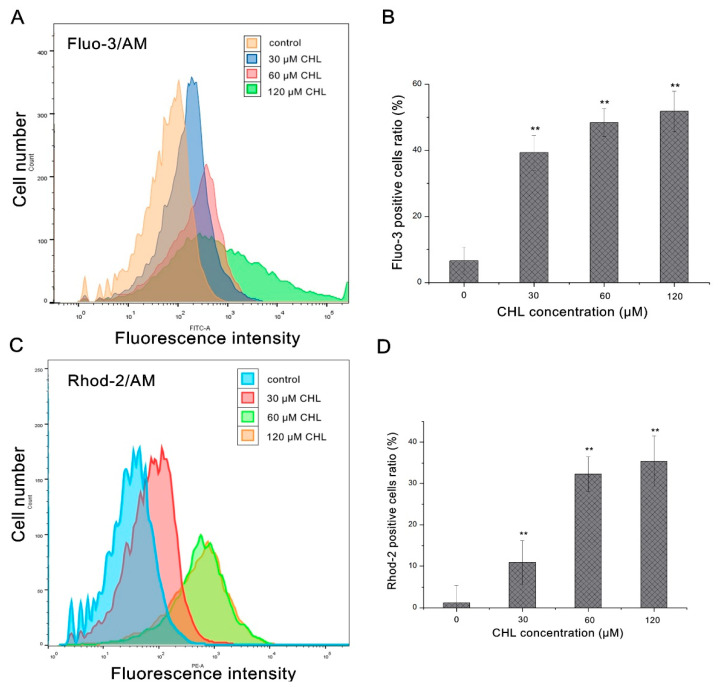
Analysis of cytosolic and mitochondrial Ca^2+^ levels in HepG2 cells after treatment with CHL. (**A**,**C**): Analysis of Fluo-3 and Rhod-2 staining by flow cytometry. (**B**): Quantification levels of Fluo-3 fluorescence. (**D**): Quantification levels of Rhod-2 fluorescence. ** *p* ≤ 0.01 vs. the negative control.

The mPTP is a non-selective pore; it’s opening may cause MMP collapse and induce cell death pathways [10]. As Ca^2+^ overload in the mitochondrial matrix is the initial mechanism of mPTP opening [11], we then investigated the variation in the Ca^2+^ concentration in cytosol and mitochondria with a cytoplasmic Ca^2+^ indicator dye (Fluo-3AM) and mitochondrial Ca^2+^ indicator dye (Rhod-2AM). As Figure 4 showed, CHL increased the mitochondrial and cytosolic Ca^2+^ levels in a dose-related manner in HepG2 cells, further confirming that mitochondrial membrane potential collapse was induced by CHL through intracellular Ca^2+^ overload in HepG2 cells.

### 2.3. Apoptosis in HepG2 Cells

**Figure 5:**
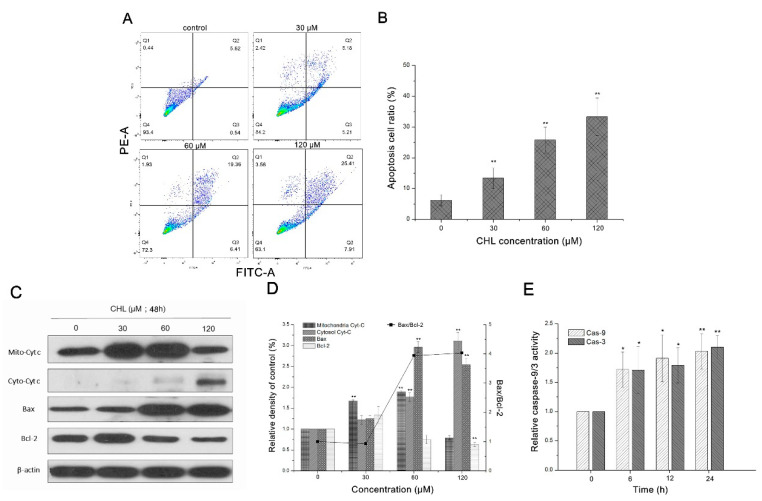
Analysis of apoptotic effects of HepG2 cells after treatment with CHL. (**A**): Analysis of Annexin V/PI staining by flow cytometry. (**B**): Quantification of apoptotic cells. (**C**): Expression of apoptotic proteins by Western blot. (**D**): Quantification levels of apoptotic proteins. (**E**): Evaluation of caspase-3/9 activity by spectrophotometric analysis. ** *p* ≤ 0.01 and * *p* ≤ 0.05 vs. the negative control.

As shown in Figure 5A, the number of apoptotic (cells in Q2) and early apoptotic cells (cells in Q3) increased gradually after treatment with various concentrations of CHL within the range of 0–120 μM. Compared to the control group (6.16 ± 1.8%), the ratio of apoptotic cells increased to 33.32 ± 6.1% following treatment with 120 μM CHL (Figure 5B). The results showed that apoptosis in HepG2 cells was induced by CHL in a dose-dependent manner.

### 2.4. Expression of Apoptosis-Related Proteins in CHL-Treated Cells

As shown in Figure 5C,D, cytochrome c (cyt-c), which is the key element of the mitochondria-mediated apoptosis pathway [12], decreased in the mitochondrial fractions after CHL treatment. In contrast, cyt-c accumulation was detected in cytosolic fractions. The proapoptotic protein Bax induces cyt-c release to the cytoplasm, and the anti-apoptotic protein Bcl-2 suppresses cyt-c and prevents the collapse of the mitochondrial membrane potential [13]. Therefore, the expression levels of Bax and Bcl-2 were detected. Figure 5C,D show that CHL treatment upregulated the expression of Bax while it downregulated Bcl-2. Moreover, the activity of the apoptosis effector caspase-3 and its precursor, caspase-9, increased in a dose-dependent manner following CHL treatment (Figure 5E). These results indicate that CHL-induced mitochondria-dependent apoptotic pathways are associated with the caspase cascade.

### 2.5. CHL Induced Autophagy in HepG2 Cells

**Figure 6:**
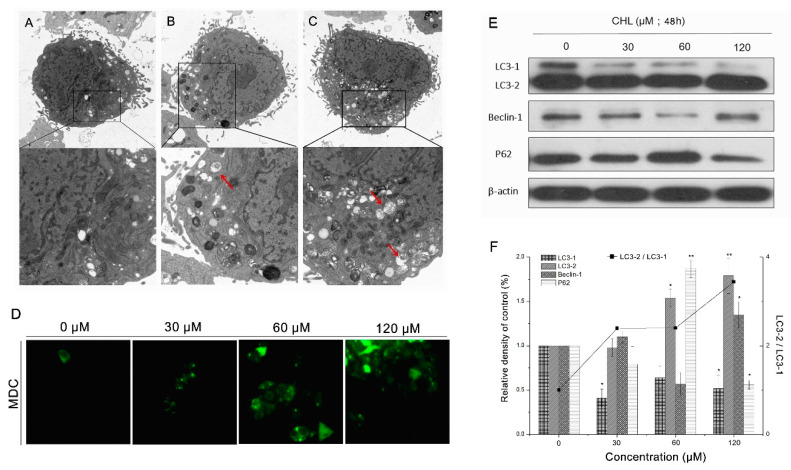
Analysis of autophagic effects of HepG2 cells after treatment with CHL. Observation of autophagy characteristics in HepG2 cells via TEM. (**A**): Control cells with normal mitochondria in well shape. (**B**,**C**): 30 μM (**B**) or 60 μM (**C**) CHL-treated cells with autophagic vacuoles (red arrows). (**D**): Analysis of MDC staining by epifluorescence (200×). (**E**): Expression of autophagy-associated proteins by Western blot. (**F**): Quantification levels of autophagy-associated proteins. ** *p* ≤ 0.01 and * *p* ≤ 0.05 vs. the negative control.

The typical characteristics of autophagy were observed by transmission electron microscopy. Figure 6A shows that after exposure to CHL, large autophagic vacuoles containing cellular material or membrane structures were detected in HepG2 cells compared with controls (Figure 6B,C). Furthermore, MDC, an autofluorescent dye, was used for monitoring autophagosome formation. As revealed in Figure 6D, CHL enhanced the fluorescence intensity of MDC on cells in a dose-related manner.

**Figure 7:**
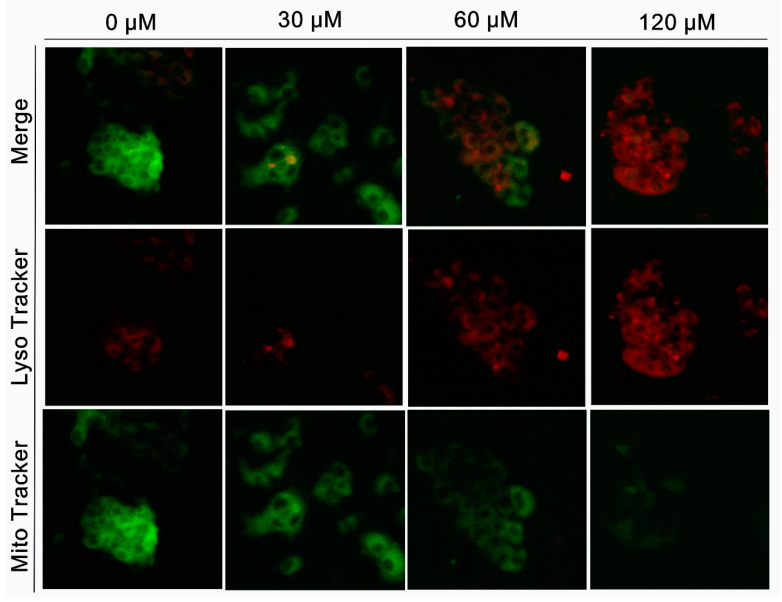
Analysis of the quantity of mitochondria in HepG2 cells after treatment with CHL. The change trends of Mito-tracker and Lyso-tracker’s fluorescence intensity were detected by epifluorescence (200×).

The expression levels of autophagy marker proteins, including Beclin-1, LC3-II/I, and p62, were determined using Western blotting. As shown in Figure 6E,F, from the overall trend, CHL upregulated the expression level of Beclin-1 and downregulated P62. In the meantime, CHL increased the conversion of LC3-I to LC3-II in HepG2 cells in a dose-dependent manner. The amounts of lysosomes and mitochondria in cells were evaluated by Lyso-tracker and Mito-tracker, respectively. The results showed that after CHL treatment, the intensity of green fluorescence had a significant decrease, while the red fluorescence intensity showed a gradual increase in a dose-dependent manner (Figure 7). These findings indicate that CHL induces autophagy in HepG2 cells, which may be associated with the damage of mitochondria.

### 2.6. CHL Induced DNA Damage and Cell Cycle Arrest

**Figure 8:**
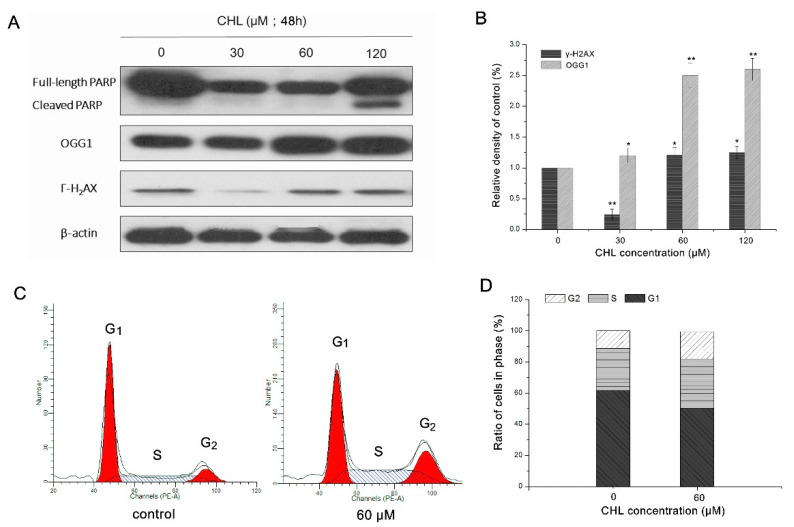
Analysis of the DNA damage and cell cycle in HepG2 cells after treatment with CHL. (**A**): Expression of PARP, γH2AX, and OGG1 by Western blot. (**B**): Quantification levels of γH2AX and OGG1. (**C**): Analysis of cell cycle by flow cytometry. (**D**), Proportion of cell cycle phases (G1, S, and G2). ** *p* ≤ 0.01 and * *p* ≤ 0.05 vs. the negative control.

After exposure to various concentrations of CHL, the HepG2 cells displayed a significantly increased level of γH2AX, which is a classic feature of DNA double-strand breaks. Moreover, the DNA mainly repaired protein PARP and OGG1, which were detected to be cleaved or accumulated in HepG2 cells, respectively (Figure 8A,B). The formation of DNA break markers and the activity of DNA-repaired proteins both indicated that CHL induced DNA damage in HepG2 cells, either directly or indirectly. In addition, the cell cycle was analyzed by flow cytometry. After 60 μM CHL treatment, the number of cells in the G1 phase decreased by 11.54%, while it increased by 4.73 and 6.18% in the S and G2 phases, respectively (Figure 8C,D). These findings indicate that the cell cycle may be arrested at the S and G2 phases after exposure to CHL.

## 3. Discussion

Recently, the safety and environmental friendliness of CHL has been gradually challenged. An increasing number of studies have indicated that CHL has acute toxic effects on non-target organisms, including humans. However, limited research has focused on the toxic effects and modes of action of CHL on humans, especially at the cellular level. In the present study, the results suggest that CHL significantly inhibited the proliferation of HepG2 cells. CHL treatment also induced mitochondrial damage, which was associated with MMP collapse, ROS accumulation, and mitochondrial calcium overload. The mitochondrial damage ultimately led to apoptosis and autophagy in HepG2 cells, which indicated that CHL inhibited cell vitality through mitochondria-mediated PCD.

Mitochondria are one of the principal vital organs in cells and have several significant functions. They are also acknowledged as the main source of ROS, mainly originating from the respiratory chain [14]. Various environmental stressors, such as pesticides, are known to enhance ROS production and disturb cellular redox homeostasis [15,16,17,18]. Our data confirmed that CHL treatment caused rapid ROS accumulation in HepG2 cells. Cells present a variety of defense mechanisms to neutralize oxidative damage. The antioxidant enzymes SOD and CAT constitute the first line of defense against superoxide radicals. However, we observed that CHL increased the ROS levels in cells and decreased the antioxidant enzyme activities of SOD and CAT. These results indicate that CHL induced oxidative stress in HepG2 cells, which was attributed to the overproduction of ROS and dysfunction of antioxidant systems. Mitochondria are highly sensitive and play a significant role in many cellular processes, including energy metabolism and PCD. Several factors contribute to mitochondrial injury, including excess ROS production, DNA damage, and exposure to toxic compounds [19,20,21,22,23]. In this study, the MMP collapse and mitochondrial Ca^2+^ overload demonstrated that oxidative stress further affected the structure or function of mitochondria.

Apoptosis, autophagy, necrosis, pyroptosis, and oncosis are the known forms of cell death. Among them, autophagy and apoptosis are specific types of PCD that can be induced by multiple irritants, such as mitochondria damage [24]. The process of apoptosis involving mitochondria is one of the main pathways known, as the mitochondrial pathway. Primitively, the released cyt-c induces Apaf-1 and triggers the emergence of apoptotic bodies that contain cyt-c, Apaf-1, and pro-caspase-9. Consequently, activated caspase-9 leads to the activation of the apoptosis effector caspase-3, which results in cell death [25]. In this study, we confirmed that CHL can induce apoptosis in HepG2 cells via the mitochondrial pathway involving increased cytosolic cyt-c and activated caspase-3.

Autophagy is a significant process for both killing stressed cells and protecting against the degradation of cytosolic proteins and organelles in a highly conserved catabolic pathway [26]. Dead cells can also be attributed to excessive autophagy. Mitochondrial ROS production and the oxidation of mitochondrial lipids are known to play a pivotal role in autophagy [27]. In mammalian cells, starvation-induced autophagy is associated with mitochondrially generated ROS such as H_2_O_2_ and O_2_^•−^. In this paper, the autophagic effect was confirmed by the observation of autophagosomes and detection of autophagy-related proteins such as LC3, P62, and Beclin-1. The transformation of LC3-I and LC3-II has been recognized as a marker of autophagy. Beclin-1 is required for the nucleation of autophagosomes, and its integration into the pre-autophagosome is significant in autophagy initiation and progression. By linking ubiquitinated substrates, p62 operates as a selective autophagy receptor for the degradation of ubiquitinated substrates in autolysosomes [28]. Once autophagy occurs, p62 is degraded. In this study, the conversion of LC3-I to LC3-II, upregulation of Beclin-1, and downregulation of p62 were observed after CHL treatment, which confirmed the occurrence of autophagy in HepG2 cells. As noted, CHL could induce mitochondrial damage. Whether these mitochondria are selected separately and cleaned through the autophagic process of mitophagy remains unclear [29]. In this study, except for the determination of the autophagy process, decreased numbers of mitochondria also were discovered. However, further evidence for these mitochondria being cleared by the mitophagy pathway is required.

In addition, oxidative stress caused by excess intracellular ROS may also induce oxidative DNA damage [30]. ROS-mediated oxidative DNA damage primarily involves base lesions and DNA strand breaks. γH_2_AX and OGG1 are viewed as markers of DNA double-strand breaks and DNA oxidative damage repair proteins, respectively. In our study, the accumulation of γH_2_AX and OGG1 in HepG2 cells after exposure to CHL was observed. These results confirmed that CHL induced DNA damage in HepG2 cells, which may be a consequence of CHL-induced oxidative stress. Cell cycle checkpoints (G1, S, and G2) serve as regulatory systems to interrupt cell cycle progression when genome damage is detected [31]. Cell cycle arrest also contributes to the repair of DNA damage. PARP can be considered a DNA damage sensor and responds to DNA single- and double-strand breaks [32]. In this study, cleaved PARP was detected, indicating that DNA damage triggered by CHL could not be repaired due to PARP loss of activity. Accordingly, the effects of CHL in the HepG2 cell cycle were examined. The results suggested that the cell cycle was arrested in the S and G2 phases after treatment with CHL. These data show that CHL-induced oxidative stress leads to DNA damage and cell cycle arrest in HepG2 cells.

## 4. Materials and Methods

### 4.1. Chemicals and Reagents

Chlorfenapyr (purity of 97%) was provided by MOLBASE Shanghai Biotechnology Co., Ltd. (Shanghai, China). CHL stock solution was dissolved in DMSO and stored at 4 °C. The stock solution was diluted with the culture medium to the corresponding concentration during the test. Penicillin and streptomycin were obtained from Gibco (Grand Island, NY, USA). Phosphate-buffered saline (PBS), dimethyl sulfoxide (DMSO), fetal bovine serum (FBS), rhodamine 123 (Rh 123), Dulbecco’s modified eagle medium (DMEM), and thiazolyl blue tetrazolium bromide (MTT) were obtained from Sigma (St. Louis, MO, USA). The Caspase-3/9 assay kit, ROS detection kit, Annexin V/PI apoptosis detection kit, and JC-1 assay kit were obtained from Keygen Biotech Co., Ltd. (Nanjing, China). The superoxidedismutase (SOD) and catalase (CAT) assay kits were all obtained from Beyotime Biotechnology (Shanghai, China). The antibodies LC-3, Bcclin-1, p62, cytochrome-c, Bcl-2, Bax, PARP, OGG1, γH2AX, beta-actin, and the secondary antibody were obtained from Servicebio Co., Ltd. (Wuhan, China). The cell mitochondria isolation kit, Monodansylcadaverine (MDC), Fluo-3 AM, Rhod-2 AM, Lyso-tracker, and Mito-tracker were obtained from Beyotime Biotechnology (Shanghai, China).

### 4.2. Cell Culture

The HepG2 cell line is derived from human liver tissue. The cell line was obtained from the China Center for Typical Culture Collection (CCTCC). The HepG2 cell line was cultured in accordance with a reported method [33]. In brief, cells were maintained in Dulbecco’s modified eagle medium (DMEM) supplemented with 10% fetal bovine serum, 100 units/mL penicillin, and 100 mg/mL streptomycin in a humidified atmosphere at 5% CO_2_ and 37 °C. The cell line was cultured in a 10 cm cell culture dish and passaged every 3 days.

### 4.3. Measurement of Cytoactivity

Tetrazolium salts such as MTT (3-(4,5-dimethylthiazol-2-yl)-2,5-diphenyltetrazolium bromide) are metabolized by mitochondrial dehydrogenases to form a blue formazan dyeand are therefore useful for the measurement of HepG2 cytotoxicity [34]. Cells with a density of 1 × 10^5^ cells per milliliter (100 μL) were seeded into each well of 96-well microplates. After overnight incubation, the cells were exposed to CHL with various concentrations (50, 100, 150, 200 and 250 μM) for different lengths of time (24, 48 and 72 h). After treatment, MTT was added with final concentration 5 mg/mL, and the cells were incubated for another 4 h. Formed formazan crystals dissolved in DMSO, and the absorbance was measured at 490 nm with an ELISA microplate reader (BioTek, Vermont, USA). The cell proliferation inhibition rate was calculated according to the calculation formula: (OD_control_ − OD_treat_)/OD_control_ × 100%.

### 4.4. Measurement of Reactive Oxygen Species (ROS) and Antioxidant Enzyme Activity

The cells were exposed to CHL for 48 h at concentrations of 30 μM (1/2 × IC_50_), 60 μM (IC_50_), and 120 μM (2 × IC_50_). The 0.1% DMSO treatment served as a control. Then, we employed detailed operation methods described in previous reports [35]. In brief, an ROS detection kit was used to measure intracellular reactive oxygen species generation. Cells were harvested by centrifugation at 2000 rpm for 3 min and washed twice with PBS. Briefly, cells were incubated with 1 mL PBS containing 10 μM DCFH-DA at 37 °C for 30 min to allow the diffusion of the fluorescent probe into the cells and its subsequent hydrolysis to non-fluorescent DCFH under the action of intracellular esterases. Then, it oxidized by ROS to DCF and was detected by flow cytometry or fluorescence microscopy. The trend of DCF fluorescence intensity was observed by a fluorescence microscope. Intracellular ROS generation was measured by a flow cytometer. The statistics of increased ROS were calculated by the ascending rate of green fluorescence (DCF fluorescence positive cell ratio) using Flowjo (V10) software (Tree Star, San Carlos, CA, USA). The activity of superoxide dismutase (SOD) and catalase (CAT) was evaluated using assay kits. After treatment with CHL at concentrations of 30, 60, and 120 μM and 0.1% DMSO (control) for 48 h, cells were harvested and the supernatants were collected. The protein concentration was determined by the BCA protein assay. Briefly, the SOD activity was measured using its ability to inhibit the reduction of WST-8, according to the instructions of the manufacturer. SOD activity was monitored spectrophotometrically at 450 nm using a microplate reader. CAT was detected by measuring the absorbance of the red compound (N-(4-anti-pyryl)-3-chloro-5-sulfonate-p-benzoquinonemonoimine) reacted by hydrogen peroxide and oxygen at 520 nm.

### 4.5. Measurement of Mitochondrial Membrane Potential (MMP)

The Rh123 and JC-1 assay kits were used to quantify the mitochondrial transmembrane potential [36]. Cells were seeded in six-well plates (1 × 10^5^ cells/mL) with 2 mL of medium and were exposed to CHL for 48 h with 30, 60, and 120 μM (0.1% DMSO for controls). Then, the cells were harvested and washed twice with PBS and then incubated with Rh123 (5 μM final concentration) and JC-1 (2 μM final concentration) in the dark for 20 min at 37 °C. After being washed twice with PBS to remove the extracellular fluorescent probe, cells were collected by centrifugation (2000 rpm for 3 min). The fluorescence trends of change were measured by a fluorescence microscope The fluorescence intensity of JC-1 aggregate (red) and JC-1 monomer (green) was measured by means of flow cytometry. The statistics of the loss of mitochondrial transmembrane potential were calculated by the decrease rate of red fluorescence vs. control using Flowjo software.

### 4.6. Measurement of Mitochondrial and Cytosolic Ca^2+^ Levels

The cells were treated with different concentrations of CHL (30, 60 and 120 μM) (0.1% DMSO for controls) for 48 h. Then, the cytosolic Ca^2+^ level (Fluo-3AM-stained cells) and mitochondrial Ca^2+^ level (Rhod-2AM-stained cells) were analyzed by a flow cytometer. The detailed operation methods are described in previous reports [37]. The harvested cells were incubated in 1 μM Fluo-3AM and 2 μM Rhod-2AM at 37 °C for 30 min, respectively. Then, the stained cells were washed with Hank’s buffer (without Ca^2+^ or Mg^2+^) and further incubated with PBS at 37 °C for 30 min before analysis by a flow cytometer. The statistics of increased Ca^2+^ levels were calculated by the red and green fluorescence intensity using Flowjo software.

### 4.7. Measurement of Apoptosis

Cell apoptosis was detected with an Annexin V-EGFP/PI apoptosis detection kit, according to the instructions of the manufacturer [38]. Briefly, cells were seeded in six-well plates (1 × 10^5^ cells/mL) with 2 mL of medium. Then, the cells were exposed to CHL for 48 h with 30, 60 and 120 μM (0.1% DMSO for controls). Cells were harvested, centrifuged at 2000 rpm for 3 min, and washed twice with PBS. The cells were then labelled with Annexin V-EGFP and PI for 15 min in the dark before analysis. Finally, cells were observed by means of flow cytometry (BD FACS Calibur) and the apoptosis rate analyzed by Flowjo software.

### 4.8. Measurement of Caspase-9/3 Activity

The cells were seeded in six-well plates (1 × 10^5^ cells/mL) with 2 mL of medium and were exposed to CHL for various times (6, 12 and 24 h) with 60 μM (0.1% DMSO for controls). Then, the activity of caspase-3 and caspase-9 was evaluated on the basis of spectrophotometric detection with the caspase-3 and caspase-9 assay kits. Cells were collected by centrifugation at 10,000 rpm for 10 min at 4 °C and were subsequently resuspended in cell lysis buffer and incubated on ice for 1 h. The resultant supernatant (50 μL) was mixed in 2 × Reaction Buffer (50 μL) and caspase-3 or caspase-9 substrate (5 μL) and then incubated at 37 °C for 4 h. Finally, the caspase-3 and caspase-9 activity was determined at 405 nm using a microplate reader [38].

### 4.9. Measurement of Morphological Observation

The cells were exposed to CHL for 48 h at different concentrations (30 and 60 μM) (0.1% DMSO for controls). The ultrastructure of cells was analyzed by transmission electron microscopy. Cells were harvested at the indicated times, rinsed twice with PBS, and cell pellets were fixed overnight at 4 °C in 2.5% glutaraldehyde in PBS. After rinsing with PBS, the cells were postfixed in 1% osmium tetroxide (OsO4) in PBS for 45 min and then washed thrice in the same buffer. The fixed cells were dehydrated in an ascending series of acetone solutions and embedded in Epon 812 (Fluka, Buchs, Switzerland). Ultrathin sections were stained with uranyl acetate and lead citrate for observation under a JEOL JEM-2100 transmission electron microscope (200 kV) (Tokyo, Japan) [33].

### 4.10. Measurement of Autophagy

The formation of autophagosome was detected by monodansylcadaverine (MDC) dye. Moreover, the quantities of mitochondria and lysosomes were observed by Mito-tracker and Lyso-tracker, respectively. We employed detailed operation methods described in previous reports [33]. In brief, cells were exposed to CHL for 48 h at different concentrations (30, 60 and 120 μM) (0.1% DMSO for controls). Then, the dye MDC, Mito-tracker, and Lyso-tracker were used to stain cells, respectively. Cells were washed twice with PBS and were incubated with MDC (1 μg/mL), Mito-tracker (0.5 μM), and Lyso-tracker (0.5 μM) for 30 min in the dark. Then, the stained cells were washed with PBS twice and observed by a fluorescence microscope (DM3000, Leica, Germany).

### 4.11. Measurement of Western Blotting

The cells were seeded in six-well plates (1 × 10^5^ cells/mL) with 2 mL of medium and were exposed to CHL for 48 h with 30, 60 and 120 μM (0.1% DMSO for controls). The total proteins and mitochondrial proteins were extracted using cold RIPA lysis buffer with 1 mM PMSF. Moreover, the protein concentration was determined using the BCA method. The cell mitochondria isolation kit was used to obtain mitochondria separately. Then, 30 μg of protein sample was segregated by 15% SDS-PAGE and transferred to a PVDF membrane using electrophoresis. The blots blocked in Tris-buffered saline–Tween with 5% non-fat dry milk at room temperature for 1 h were then treated for immunoblotting with primary antibodies and the HRP-conjugated secondary antibodies. The immune-reactive proteins were visualized using the Super Signal West Pico Trial Kit. The density of each band was quantified using Image Lab software (BioRad, California, USA) and normalized to its respective loading control. The final data were expressed as the ratio of the intensity of the protein in treated cells to that of the corresponding protein in control cells [39].

### 4.12. Measurement of Cell Cycle

The cells were exposed to CHL with 60 μM for 24 h (0.1% DMSO for controls). The detailed operation methods are described previous reports [40]. The cell cycle was determined by flow cytometry analysis of propidium iodide (PI)-stained cells. In brief, cells were trypsinized, washed with cold PBS, and fixed in ice-cold 70% ethanol for 1 h. Then, the cells were resuspended with 2 mL PBS (supplemented with 10 μL of RNase) and incubated at 37 °C for 30 min. After this, the cells were stained with 1 mg/mL PI before analysis by flow cytometry. The data were processed using FlowJo software.

### 4.13. Statistical Analysis

All experiments in this study were performed at least three times. The results were calculated as the mean ± standard error of the mean (SEM). The statistical analysis used SPSS V18 and Excel 2013. Statistical significance was determined by ANOVA and Student’s *t* test (** *p* ≤ 0.01, * *p* ≤ 0.05).

## 5. Conclusions

In conclusion, CHL could inhibit proliferation through mitochondria-mediated PCD and cell cycle arrest. On one hand, CHL treatment induced mitochondrial damage, which is associated with ROS accumulation and mitochondrial calcium overload, ultimately leading to apoptosis and autophagy in HepG2 cells. On the other hand, CHL-induced oxidative stress led to DNA damage and cell cycle arrest in HepG2 cells. The findings place a focus on the latent human health threats posed by CHL. Moreover, the study provides a theoretical basis for the toxic effects and mode of action of CHL on humans, which may contribute to the treatment of CHL poisoning.

## Data Availability

Not applicable.

## References

[1] Wang X., Wang J., Cao X., Wang F., Yang Y., Wu S., Wu Y. (2019). Long-term monitoring and characterization of resistance to chlorfenapyr in *Plutella xylostella* (Lepidoptera, Plutellidae) from China. Pest Manag. Sci..

[2] Zhao Y., Wang Q., Yao W., Zhang Z., Wei M. (2017). Chlorfenapyr, a potent alternative insecticide of phoxim to control *Bradysia odoriphaga* (Diptera, sciaridae). J. Agric. Food Chem..

[3] Albers P., Klein P., Green D., Melancon M., Bradley B., Noguchi G. (2006). Chlorfenapyr and mallard ducks, overview, study design, macroscopic effects, and analytical chemistry. Environ. Toxicol. Chem..

[4] Chen X., Zheng J., Teng M., Zhang J., Qian L., Duan M., Zhao F., Zhao W., Wang Z., Wang C. (2021). Bioaccumulation, metabolism and the toxic effects of chlorfenapyr in zebrafish (*Danio rerio*). J. Agric. Food Chem..

[5] Shao Y., Xin X., Liu Z., Wang J., Gui Z. (2021). Transcriptional response of detoxifying enzyme genes in *Bombyx mori* under chlorfenapyr exposure. Pestic. Biochem. Phys..

[6] Shi W., Shi W., Gao S., Lu Y., Cao Y., Zhou P. (2010). Effects of nanopesticide chlorfenapyr on mice. Toxicol. Environ. Chem..

[7] Baek B., Kim S., Yoon W., Heo T., Yun Y., Kang H. (2016). Chlorfenapyr-induced toxic leukoencephalopathy with radiologic reversibility, a case report and literature review. Korean J. Radiol..

[8] Periasamy S., Deng J., Liu M. (2016). Who is the real killer? Chlorfenapyr or detergent micelle-chlorfenapyr complex?. Xenobiotica.

[9] Tharaknath V., Prabhakar Y., Kumar K., Babu N. (2013). Clinical and radiological findings in chlorfenapyr poisoning. Ann. Indian Acad. Neur..

[10] Sedlic F., Sepac A., Pravdic D., Camara A., Bienengraeber M., Brzezinska A., Wakatsuki T., Bosnjak Z. (2010). Mitochondrial depolarization underlies delay in permeability transition by preconditioning with isoflurane, roles of ROS and Ca^2+^. Am. J. Physiol.-Cell. Physiol..

[11] Hurst S., Hoek J., Sheu S. (2017). Mitochondrial Ca^2+^ and regulation of the permeability transition pore. J. Bioenerg. Biomembr..

[12] Granville D., Cassidy B., Ruehlmann D., Choy J., Brenner C., Kroemer G., Breemen C., Margaron P., Hunt D., Mcmanus B. (2001). Mitochondrial release of apoptosis-inducing factor and cytochrome c during smooth muscle cell apoptosis. Am. J. Pathol..

[13] Yip K., Reed J. (2008). Bcl-2 family proteins and cancer. Oncogene.

[14] Schrauwen P., Hesselink M. (2004). Oxidative capacity, lipotoxicity, and mitochondrial damage in type 2 diabetes. Diabetes.

[15] Yang M., Hao Y., Gao J., Zhang Y., Xu W., Tao L. (2017). Spinosad induces autophagy of *Spodoptera frugiperda* Sf9 cells and the activation of AMPK/mTOR signaling pathway. Comp. Biochem. Physiol. C.

[16] Yao T., Li H., Ren Y., Feng M., Hu Y., Yan H., Peng L. (2022). Extraction and recovery of phenolic compounds from aqueous solution by thermo-separating magnetic ionic liquid aqueous two-phase system. Sep. Purif. Technol..

[17] Yang Y., Gao J., Zhang Y., Xu W., Hao Y., Xu Z., Tao L. (2018). Natural pyrethrins induce autophagy of HepG2 cells through theactivation of AMPK/mTOR pathway. Environ. Pollut..

[18] Luo W., Wang J., Wang Y., Tang J., Ren Y., Geng F. (2021). Bacteriostatic effects of high-intensity ultrasonic treatment on Bacillus subtilis vegetative cells. Ultrason. Sonochem..

[19] Sheehan J., Swerdlow R., Miller S., Davis E., Parks J., Parker W., Tuttle J. (1997). Calcium homeostasis and reactive oxygen species production in cells transformed by mitochondria from individuals with sporadic alzheimer’s disease. J. Neurosci..

[20] Yang M., Wang B., Gao J., Zhang Y., Xu W., Tao L. (2017). Spinosad induces programmed cell death involves mitochondrial dysfunction and cytochrome C release in *Spodoptera frugiperda* Sf9 cells. Chemosphere.

[21] Ren Y., Xia H., Lu L., Zhao G. (2021). Characterization of the complete chloroplast genome of *Hordeum vulgare* L var *trifurcatum* with phylogenetic analysis. Mitochondrial DNA B.

[22] Li Q., Ren Y., Shi X., Peng L., Zhao J., Song Y., Zhao G. (2019). Comparative mitochondrial genome analysis of two ectomycorrhizal fungi (*Rhizopogon*) reveals dynamic changes of intron and phylogenetic relationships of the subphylum agaricomycotina. Int. J. Mol. Sci..

[23] Zhang Y., Chang Y., Cao H., Xu W., Li Z., Tao L. (2018). Potential threat of Chlorpyrifos to human liver cells via the caspase-dependent mitochondrial pathways. Food Agric. Immunol..

[24] Elmore S. (2007). Apoptosis, a review of programmed cell death. Toxicol. Pathol..

[25] Johnson V., Ko S., Holmstrom T., Eriksson J., Chow S. (2000). Effector caspases are dispensable for the early nuclear morphological changes during chemical-induced apoptosis. J. Cell. Sci..

[26] Lionaki E., Markaki M., Palikaras K., Tavernarakis N. (2015). Mitochondria, autophagy and age-associated neurodegenerative diseases, new insights into a complex interplay. BBA-Bioenerg..

[27] Lee J., Giordano S., Zhang J. (2012). Autophagy, mitochondria and oxidative stress, cross-talk and redox signalling. Biochem. J..

[28] Itakura E., Mizushima N. (2011). P62 targeting to the autophagosome formation site requires self-oligomerization but not LC3 binding. J. Cell Biol..

[29] Lemasters J. (2005). Selective mitochondrial autophagy, or mitophagy, as a targeted defense against oxidative stress, mitochondrial dysfunction, and aging. Rejuvenation Res..

[30] Xu W., Yang M., Gao J., Zhang Y., Xu W., Tao L. (2017). Oxidative stress and DNA damage induced by spinosad exposure in *Spodoptera frugiperda* Sf9 cells. Food Agric. Immunol..

[31] Tamar E., Chris N. (1995). Cellular responses to DNA damage, cell-cycle checkpoints, apoptosis and the roles of p53 and ATM. Trends Biochem. Sci..

[32] Celik-Ozenci C., Tasatargil A., Tekcan M., Sati L., Gungor E., Isbir M., Demir R. (2011). Effects of abamectin exposure on male fertility in rats, potential role of oxidative stress-mediated poly (ADP-ribose) polymerase (PARP) activation. Regul. Toxicol. Pharmacol..

[33] Zhang Y., Guo W., Gao J., Xu Z., Tao L., Zhong L., Xu W. (2019). Spinetoram confers its cytotoxic effects by inducing AMPK/MTOR-mediated autophagy and oxidative DNA damage. Ecotoxicol. Environ. Saf..

[34] Ren Y., Li Q., Lu L., Jin H., Tao K., Hou T. (2021). Toxicity and physiological actions of biflavones on potassium current in insect neuronal cells. Pestic. Biochem. Phys..

[35] Ren Y., Mu Y., Yue Y., Jin H., Tao K., Hou T. (2019). Neochamaejasmin A extracted from Stellera chamaejasme L. induces apoptosis involved mitochondrial dysfunction and oxidative stress in Sf9 cells. Pestic. Biochem. Phys..

[36] Ren Y., He L., Jin H., Tao K., Hou T. (2018). Cytotoxicity evaluation and apoptosis-inducing effects of furanone analogs in insect cell line SL2. Food Agric. Immunol..

[37] Ren Y., Yang N., Yue Y., Jin H., Tao K., Hou T. (2018). Investigation of novel pyrazole carboxamides as new apoptosis inducers on neuronal cells in *Helicoverpa zea*. Bioorg. Med. Chem..

[38] Ren Y., Jin H., Tao K., Hou T. (2015). Apoptotic effects of 1,5-bis-(5-nitro-2-furanyl)-1,4-pentadien-3-one on *Drosophila* SL2 cells. Mol. Cell. Toxicol..

[39] Ren Y., Shi J., Mu Y., Jin H., Tao K., Hou T. (2019). AW1 neuronal cell cytotoxicity, the mode of action of insecticidal fatty acids. J. Agric. Food Chem..

[40] Ren Y., Li Q., Lu L., Jin H., Tao K., Hou T. (2021). Isochamaejasmin induces toxic effects on *Helicoverpa zea* via DNA damage and mitochondria-associated apoptosis. Pest Manag. Sci..

